# Gallbladder remnant: A potential source for biliary stones postcholecystectomy; a case report in a child with sickle cell disease

**DOI:** 10.1002/jpr3.12039

**Published:** 2024-01-25

**Authors:** Ahmad Miri, Shahab Abdessalam, Andria M. Powers, Ruben E. Quiros‐Tejeira, Chinenye R. Dike

**Affiliations:** ^1^ Department of Pediatrics, Division of Pediatric Gastroenterology, Hepatology and Nutrition University of Nebraska Medical Center Omaha Nebraska USA; ^2^ Pediatric Surgeon, LLC Omaha Nebraska USA; ^3^ Department of Pediatrics, Division of Pediatric Radiology University of Nebraska Medical Center Omaha Nebraska USA; ^4^ Department of Pediatrics, Division of Pediatric Gastroenterology, Hepatology and Nutrition University of Alabama at Birmingham Birmingham Alabama USA

**Keywords:** cholecystectomy, choledocholithiasis, cholelithiasis, hemolysis, pediatric

## Abstract

Stone formation in a gallbladder remnant is a rare postcholecystectomy complication. This report describes the case of gallstones in a gallbladder remnant of an adolescent with sickle cell disease (SCD) years after laparoscopic cholecystectomy. A 15‐year‐old female with SCD presented to our gastroenterology clinic with concerns of recurrent choledocholithiasis despite cholecystectomy 2 years before presentation. About 4 months before presentation to our clinic, she was evaluated at the referring physician's emergency department for recurrent severe abdominal pain of 1 month duration. After admission to the hospital, common bile duct stones were seen on magnetic resonance cholangiopancreatography (MCRP) imaging and subsequently removed via endoscopic retrograde cholangiopancreatography (ERCP). On review of her MRCP and ERCP at our hospital, a remnant of gallbladder containing multiple stones was identified. She subsequently underwent a laparoscopic resection of the gallbladder remnant. Clinicians should consider biliary duct imaging in children with biliary colic following cholecystectomy, especially those with history of chronic hemolysis.

## 1 INTRODUCTION

The prevalence of cholelithiasis in children varies between 0.13% and 2%.^1^ Sickle cell disease (SCD) is associated with intravascular hemolysis and increased incidence of pigmented gallstones. The prevalence of cholelithiasis in children with SCD is estimated to be 10%–37%.^2^ In the general population, choledocholithiasis occurs in 10%–20% of children with gallstones.^1^ In patients with SCD, the incidence of choledocholithiasis is estimated to be around 18%.^3^

Postcholecystectomy complications are multiple, and late complications include papillary stenosis, retained or recurrent common bile duct stones, incomplete resection of the gallbladder, and biliary stricture. Incomplete resection of gallbladder known as “gallbladder remnant” is rare and leads to residual pigmented stones, and may occur intentionally or unintentionally.^4^ Incomplete gallbladder removal may occur with severe cholecystitis, adhesions, excessive bleed or when the anatomy is distorted with possible risk of injury to the bile duct. It may lead to formation of gallstones in the remnant, cystic duct, or choledocholithiasis. An elevated gamma‐glutamyl transferase (GGT) was the most common laboratory abnormality seen in a case series of remnant cystic duct stones resulting from incomplete cholecystectomy.^5^ A population‐based study ^4^ showed 0.5% rate of repeat cholecystectomy from which 0.25% were analyzed. They showed a repeat cholecystectomy was likely to occur emergently and require intensive care postoperative care.

## 2 CASE

**Figure 1:**
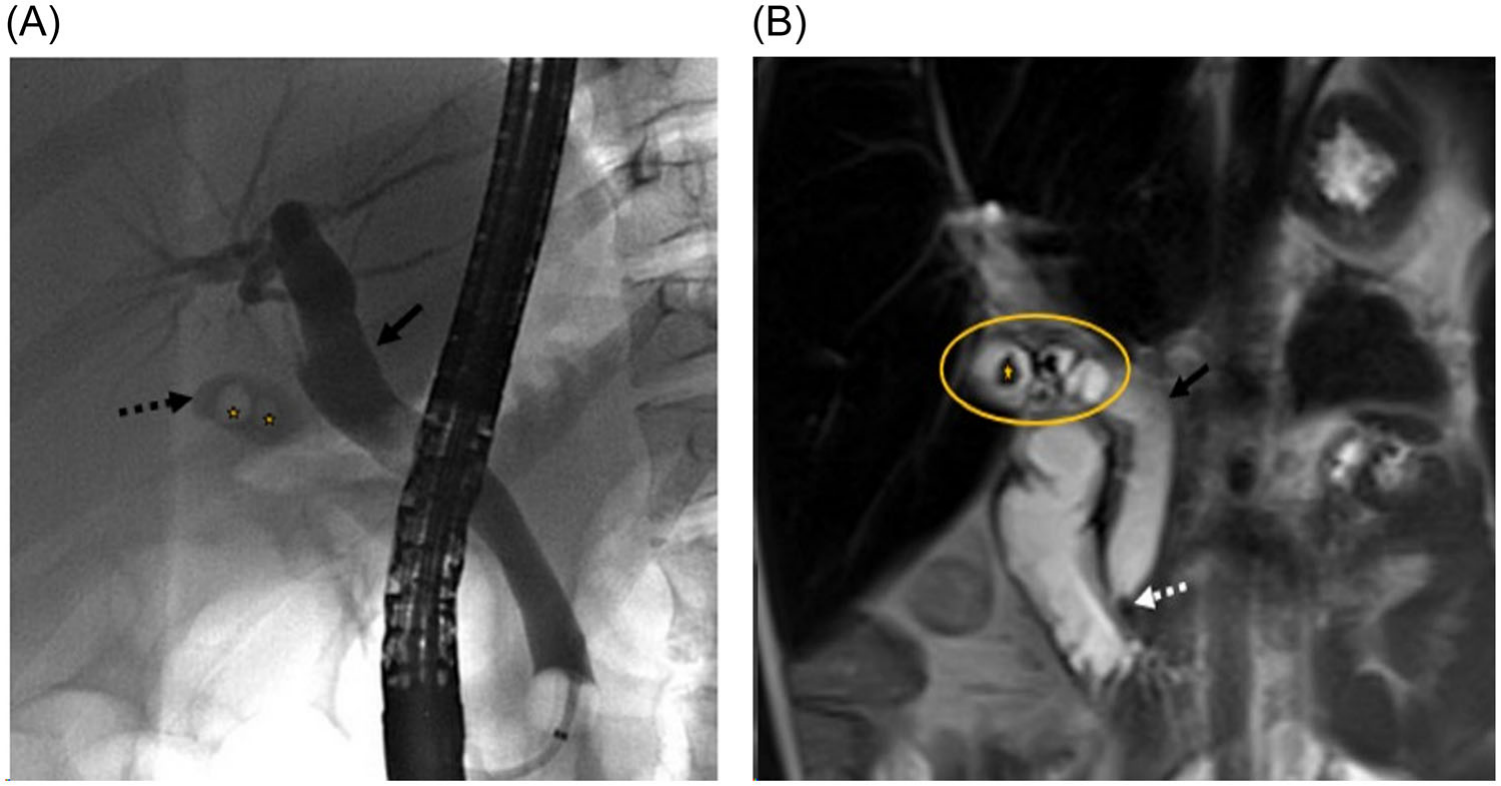
(A) Endoscopic retrograde cholangiopancreatography (ERCP) with contrast opacified biliary system. Imaging done 2 years after 1st cholecystectomy (before repeat cholecystectomy). Two lucent filling defects (starred) in the gallbladder remnant (dashed arrow). (B) Magnetic resonance cholangiopancreatography (MCRP) imaging done 2 years after 1st cholecystectomy (before repeat cholecystectomy). Cor T2‐weighted MR image shows dilated common bile duct (arrow) with hypointense calculus at the ampulla (dashed arrow). Ovoid fluid filled gallbladder remnant (circled) contains numerous additional hypointense calculi (starred).

Our patient is a 15‐year‐old female with history of SCD (HbSS genotype). She developed postprandial epigastric abdominal pain, nausea, emesis, back pain, and worsening jaundice of 1 month duration; 2 years postcholecystectomy. These symptoms prompted multiple visits to her pediatrician and emergency room. She was subsequently admitted to an outside facility due to worsening symptoms. An evaluation revealed anemia with stable hemoglobin at 9.5 g/dL. Unconjugated bilirubin of 22.5 mg/dL, AST 158 U/L, ALT 266 U/L. Ultrasound of the abdomen showed dilated common bile duct (CBD). A magnetic resonance cholangiopancreatography (MRCP) revealed a dilated CBD (14 mm), and an obstructing stone (5 mm) at the level of ampulla of Vater with no radiographic evidence of pancreatitis. She then underwent endoscopic retrograde cholangiopancreatography (ERCP) for CBD stone. After removal of CBD stone, her symptoms resolved, and transaminases and bilirubin improved. She was then referred to our pediatric gastroenterology clinic to evaluate etiology for choledocholithiasis after cholecystectomy. At that visit, she reported complete resolution of her symptoms after the ERCP. Physical exam was unremarkable except for mild scleral icterus. Her abdomen was nontender without any palpable hepatosplenomegaly. Work up showed hemoglobin at baseline of 10 g/dL, Reticulocyte count of 18%, no leukocytosis, AST 83 U/L, ALT 39 U/L, GGT 44 U/L, unconjugated bilirubin 3.6 mg/dL, conjugated bilirubin was 0 mg/dL. Work up for mildly elevated transaminases which were done concurrently with the repeat liver panel revealed normal alpha 1 Antitrypsin phenotype (MM), total lgG, ceruloplasmin, and negative hepatitis B surface Ag, hepatitis A, and Hepatitis C antibodies. Review of the referral facility MRCP and ERCP images revealed a dilated cystic duct and a remnant of gallbladder containing multiple stones (Figure 1). Per initial operation report of laparoscopic cholecystectomy that was completed over 2 years ago, the inflammation around the porta was extensive making it difficult to identify the cystic duct, so the gallbladder was removed as low as possible with a stapler to avoid any potential injury to the CBD. The size of remaining gallbladder tissue left after that initial laparoscopic cholecystectomy completed over 2 years ago was not well appreciated at the time of that surgery.

**Figure 2:**
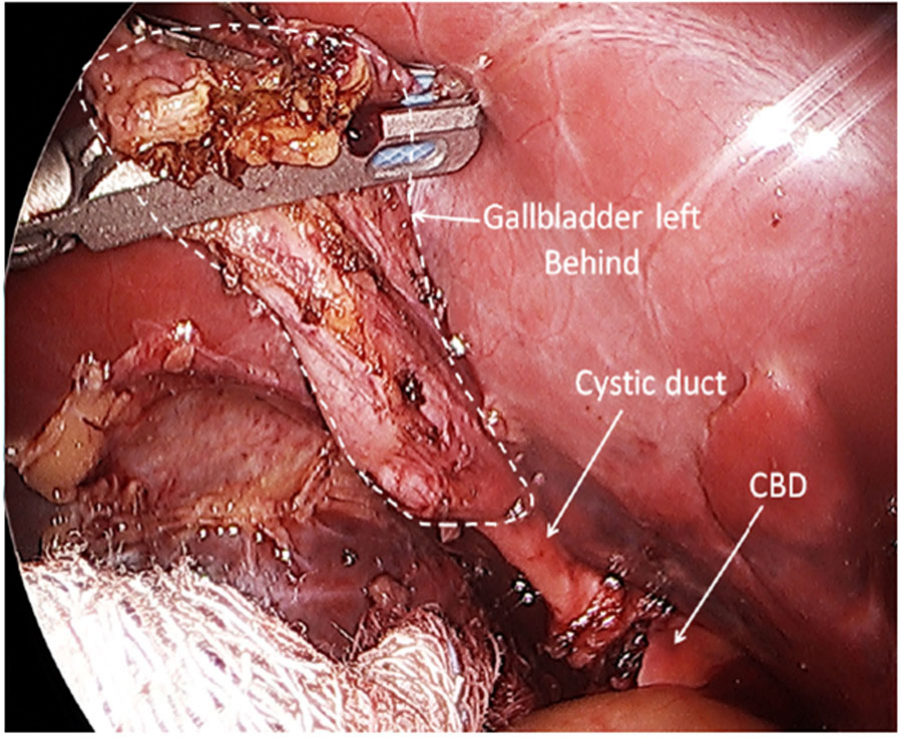
Intraoperative image shows remnant of patient's gallbladder during laparoscopic resection of remnant of gallbladder. This was taken during the recent repeat laparoscopic cholecystectomy before resection of the gallbladder remnant. CBD, common bile duct.

She subsequently underwent a successful laparoscopic resection of the gallbladder remnant (Figure 2) with improvement in her transaminases (AST: 54 U/L, ALT: 36 U/L and unconjugated bilirubin decreased to her baseline levels of 2.2 mg/dL. GGT was not repeated after surgery since it was normal at 28 U/L a few days before surgery.

## 3 DISCUSSION

SCD is associated with unconjugated hyperbilirubinemia due to red blood cell hemolysis with increased risk of cholelithiasis and choledocholithiasis. Pigmented stones are usually small in size and can cause low‐grade bile duct obstruction as they move easily from gallbladder to bile ducts.^6^ Gallstones are formed from the supersaturation of bile with calcium bilirubinate, the calcium salt of unconjugated bilirubin.

Gallbladder remnant is usually an incidental finding and has been reported after both open and laparoscopic cholecystectomy especially after laparoscopic cholecystectomy.^7^

A PubMed and Cochrane literature review showed variable detection time for a gallbladder remnant ranging from 4 months to up to 25 years postcholecystectomy^8^ with most of the cases reported in adults. Our patient presented with choledocholithiasis 2 years after cholecystectomy. The review also showed that common presenting symptoms were abdominal pain, jaundice, and fever.^8^ Our patient only had mild scleral icterus at the time of attempted gallbladder remnant extirpation.

Different types of imaging and procedures are used in the diagnosis of gallbladder remnant and its complications including ultrasonography, computed tomography scan, MRCP, and ERCP.^8^ In our patient, initial abdominal ultrasound only showed dilated CBD. CBD dilation can be seen postcholecystectomy and has been reported in up to 29% of patients, 1 year postcholecystectomy.^9^ MRCP was subsequently done in our patient, and this revealed a CBD stone and gallbladder remnant.

The surgical approach for removal of a gallbladder remnant remains controversial. While some surgeons discourage the laparoscopic approach due to scar tissue formation around the Calot's triangle (or hepatocytic triangle with cystic duct, common hepatic duct, and liver margin; this triangle is a space at the porta hepatis of surgical importance which is dissected during cholecystectomy) after the initial cholecystectomy. Although, more recent studies report the laparoscopic approach as both feasible, and less invasive when performed by expert laparoscopic surgeons, a large recent study of 93 patients reported a high conversion rate of the laparoscopic approach to open cholecystectomy for gallbladder remnant.^10^ Our patient underwent a resection of a gallbladder remnant left during a prior laparoscopic cholecystectomy (over 2 years ago) via a repeat laparoscopic approach and did well without any complications postsurgery. We requested follow‐up in the GI clinic, but she did not follow up. She continues to follow in the hematology clinic and her transaminases are monitored there. She remains at risk of choledocholithiasis, and pancreatitis given her background of chronic hemolysis.

In summary, we report a 15‐year‐old with SCD who presented with choledocholithiasis; 2 years postcholecystectomy from a gallbladder remnant and to our knowledge this is the first report of gallbladder remnant in an adolescent with SCD. Symptomatic incomplete gallbladder resection is a rare late complication following cholecystectomy especially with the laparoscopic approach. A complete resection of the remnant via a laparoscopic or open approach usually results in resolution of symptoms. Our patient's gallbladder remnant was left intentionally due to risk of damage to important structures given significant inflammation at first surgery. Providers should consider further imaging such as MRCP to evaluate the biliary duct in children particularly those with chronic hemolytic disorders who present with biliary colic, obstructive jaundice, or pancreatitis following cholecystectomy. Our case also highlights the importance of performing an elective cholecystectomy in asymptomatic patients with cholelithiasis before significant gallbladder inflammation is present.

## CONFLICT OF INTEREST STATEMENT

The authors declare no conflict of interest.
